# Case Report: Necrotizing fasciitis caused by Vibrio vulnificus without recalled seafood or seawater exposure diagnosed by metagenomic next-generation sequencing

**DOI:** 10.3389/fmed.2026.1963954

**Published:** 2026-09-30

**Authors:** Caifei Yue, Bangbo Xia

**Affiliations:** Department of Emergency Medicine, Sir Run Run Shaw Hospital, Zhejiang University School of Medicine, Shaoxing, Zhejiang, China

**Keywords:** metagenomic next-generation sequencing, necrotizing fasciitis, no recalled aquatic exposure, septic shock, Vibrio vulnificus

## Abstract

Vibrio vulnificus is a highly virulent marine Gram-negative pathogen that rapidly induces soft tissue necrosis and fatal septic shock, with infections generally linked to raw seafood ingestion or open wound contact with seawater. We report an atypical severe case of V. vulnificus septic shock in a 60-year-old male patient without recalled seafood or seawater exposure and without classic underlying high-risk diseases such as liver cirrhosis and diabetes mellitus. The patient presented with rapidly progressive swelling and subcutaneous ecchymosis of the right lower limb, which quickly deteriorated into refractory septic shock within 24 h. Conventional empiric broad-spectrum antibiotics failed to halt disease progression before pathogen identification. Metagenomic next-generation sequencing (mNGS) rapidly detected V. vulnificus sequences on the second admission day, and the diagnosis was further confirmed by blood culture. Multidisciplinary interventions including repeated radical surgical debridement, vacuum sealing drainage (VSD), targeted anti-infective therapy and comprehensive organ support finally achieved complete recovery. The patient recovered normal walking function during six-month outpatient follow-up. This rare occult infection case reminds clinicians to maintain high suspicion of V. vulnificus infection for rapidly advancing necrotizing fasciitis combined with septic shock, even when no aquatic exposure is recalled. Meanwhile, this work validates the critical value of mNGS for early pathogen identification in unexplained life-threatening sepsis.

## Introduction

Vibrio vulnificus is a halotolerant marine Gram-negative bacterium mainly distributed in warm estuarine and coastal low-salinity waters. Human infection typically follows consumption of contaminated raw seafood or direct contamination of broken skin by polluted seawater (1), and the clinical spectrum ranges from mild self-limited gastroenteritis to fulminant necrotizing fasciitis and irreversible septic shock, with mortality exceeding 50% among patients with chronic liver disease, diabetes or immunodeficiency (2). In patients with chronic liver disease, particularly those with cirrhosis complicated by spontaneous bacterial peritonitis, V. vulnificus is a well-recognized cause of rapidly fatal primary septicemia; however, fulminant soft tissue infection can also occur in previously healthy patients without these risk factors, as in the present case. Global warming has progressively expanded the ecological range of V. vulnificus and increased the annual incidence of infection worldwide, making this pathogen an increasingly important cause of rapidly fatal soft tissue infection and sepsis (3).

Early diagnosis of V. vulnificus soft tissue infection, however, remains challenging because the initial skin lesions manifest only as local erythema and swelling and are easily mistaken for simple cellulitis, contributing to diagnostic delay. Conventional bacterial culture requires 48–72 h to obtain positive results and cannot meet the urgent diagnostic demands of critically ill patients whose condition may deteriorate within hours (4). The Laboratory Risk Indicator for Necrotizing Fasciitis (LRINEC) score also shows low sensitivity for V. vulnificus-associated soft tissue infection, frequently producing false-negative results that interfere with early clinical judgment (5). These limitations leave a dangerous diagnostic gap for fulminant infection. Metagenomic next-generation sequencing (mNGS) can detect pathogenic nucleic acids directly from clinical samples without culture, providing an unbiased and ultra-rapid approach that has shown particular value in the diagnosis of unexplained severe sepsis (6).

Most reported V. vulnificus infections have occurred in patients with a clear history of aquatic exposure, such as eating raw seafood or wound contact with seawater; occult infections without such exposure are rarely reported and are mostly attributed to indirect contact with contaminated aquatic products, unrecognized micro-trauma exposed to contaminated water, or rare vector-borne transmission (4). Because clinicians frequently exclude V. vulnificus when an aquatic exposure history is absent, these occult cases are prone to delayed differential diagnosis. Here we describe a patient who developed rapidly progressive necrotizing fasciitis and septic shock caused by V. vulnificus despite the patient and his cohabiting family members clearly reporting no history of seafood consumption or seawater contact and no classic underlying risk factors, aiming to enrich clinical data of atypical V. vulnificus infection and demonstrate the application value of mNGS for early diagnosis of occult fulminant infection.

## Case report

### Clinical manifestations and admission examination

A 60-year-old male patient presented to our emergency department in October 2025 (hospital day 1) with a 24-hour history of rapidly progressive erythema, swelling and subcutaneous ecchymosis of the right leg. The symptoms began with soreness and local pruritus of the calf and progressed within one day to massive limb edema, tense bullae, fatigue, chest tightness and palpitations. The patient recalled no trauma, seafood consumption or seawater contact, and reported no history of chronic hepatitis, liver cirrhosis, diabetes, autoimmune diseases or long-term immunosuppressant use. The patient resided in Cixi, Ningbo, Zhejiang Province, approximately 80 km from the nearest coastal waters, and had no occupational or recreational contact with marine environments.

On admission (hospital day 1), vital signs indicated hemodynamic instability: body temperature 37.4  °C, heart rate 136 beats/min, respiratory rate 31 breaths/min, blood pressure 93/68 mmHg, accompanied by acute delirium. Physical examination revealed diffuse swelling, patchy subcutaneous hemorrhage and scattered tense blisters filled with serosanguineous fluid covering the entire right lower limb.

Laboratory tests performed on the same day indicated severe systemic inflammatory response and multiple organ injury: white blood cell count 13.20 × 10⁹/L, procalcitonin (PCT) > 100 ng/mL, C-reactive protein (CRP) 287.8 mg/L; elevated brain natriuretic peptide, troponin I and hepatic transaminases with hyperbilirubinemia. The Laboratory Risk Indicator for Necrotizing Fasciitis (LRINEC) score calculated from these values on admission was 4. Mild hypokalemia was detected, while routine coagulation function and serological screening for human immunodeficiency virus (HIV) and hepatitis B and C viruses remained within normal reference ranges. Septic shock was confirmed, and the patient was immediately transferred to the intensive care unit (ICU) for emergency multidisciplinary rescue.

### Therapeutic interventions

Immediately upon ICU admission on the same day (hospital day 1), empiric broad-spectrum antibiotic therapy was initiated with intravenous meropenem (8 days), linezolid (2 days) and levofloxacin (5 days). Emergency bedside fasciotomy, radical debridement and vacuum sealing drainage (VSD) were performed on the right calf and popliteal fossa for soft tissue decompression (Figure 1A).

**Figure 1:**
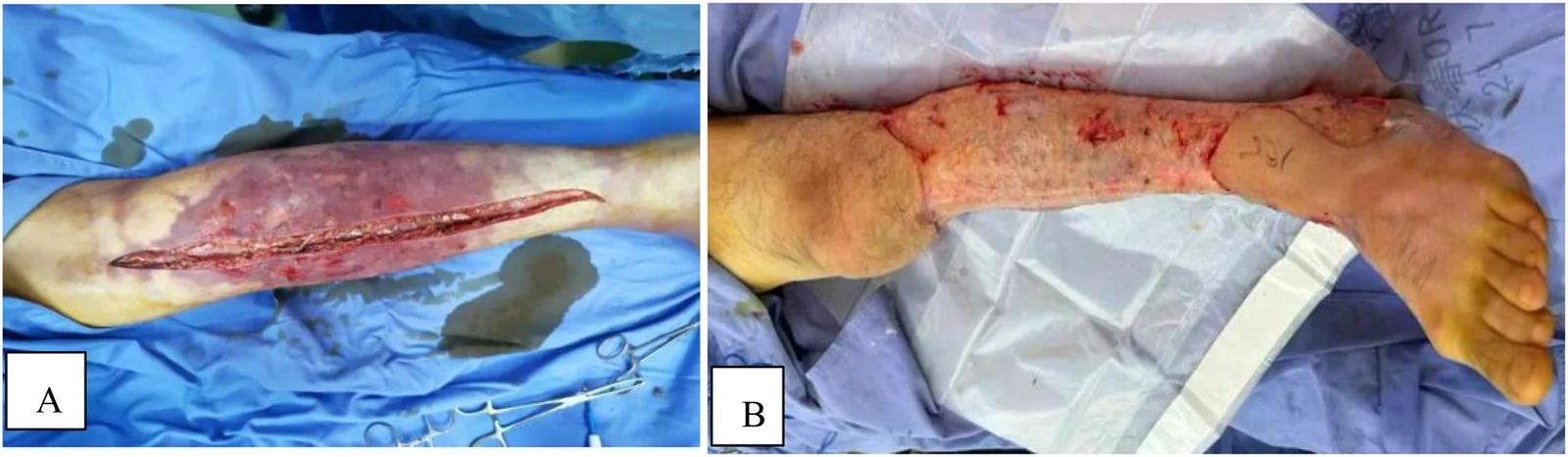
Intraoperative sequential debridement images of right lower limb necrotizing fasciitis. **(A)** Initial emergency fasciotomy with longitudinal full-layer incision to decompress swollen soft tissue and expose extensive necrotic subcutaneous tissue; **(B)** Wound appearance after secondary radical debridement, with complete resection of all non-viable tissue.

Because recurrent necrotic tissue and new bullae developed, a second thorough debridement with complete resection of all necrotic tissue was performed on hospital day 4 (Figure 1B). Vasopressor support with norepinephrine was gradually weaned off by hospital day 3, and the patient was extubated and switched to nasal cannula oxygen on hospital day 5. After continuous VSD negative-pressure management, clean granulation tissue formed on the wound bed (Figure 2A), and staged mesh split-thickness skin grafting was implemented to repair the large-area soft tissue defect (Figure 2B). Following identification of V. vulnificus, targeted anti-infective therapy with intravenous ceftazidime (2 days) was administered during the final two hospital days, and sequential surgical debridement and organ function support were maintained throughout hospitalization. The serial inflammatory markers (CRP and PCT) and the antibiotic therapy timeline are depicted in Figure 3. Sepsis was gradually controlled, and all damaged organ functions fully recovered. The patient was discharged on hospital day 9 and transferred to a local hospital for continued VSD dressing changes. At six-month telephone and outpatient follow-up, the patient regained complete normal walking ability without limb dysfunction or recurrent infection.

**Figure 2:**
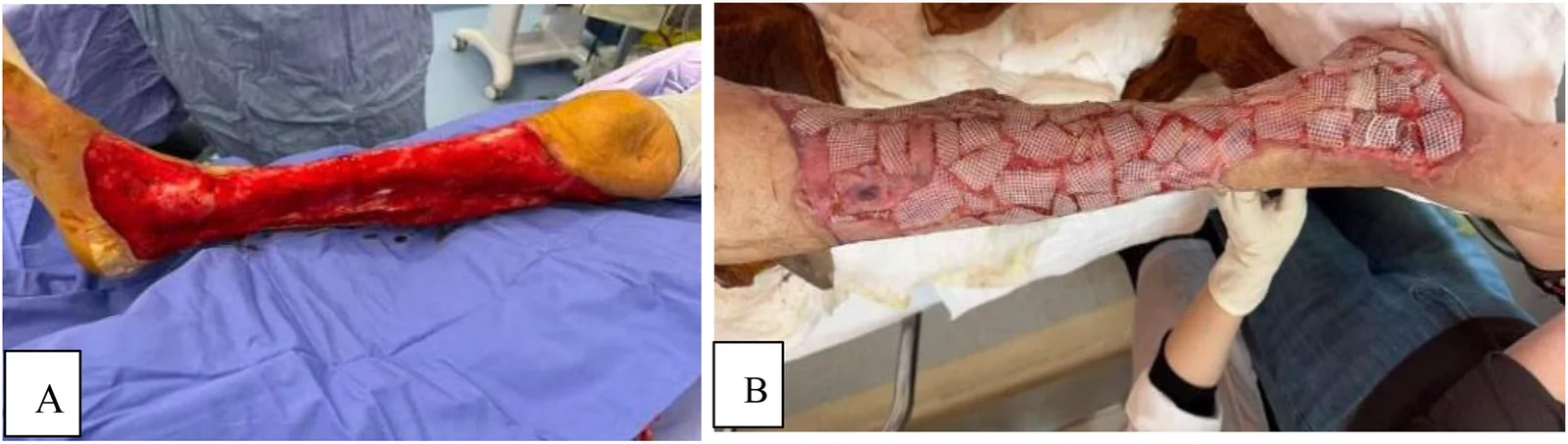
Staged soft tissue reconstructive surgery. **(A)** Clean wound bed covered with healthy granulation tissue after long-term vacuum sealing drainage; **(B)** Large soft tissue defect reconstructed with mesh split-thickness skin grafts.

**Figure 3:**
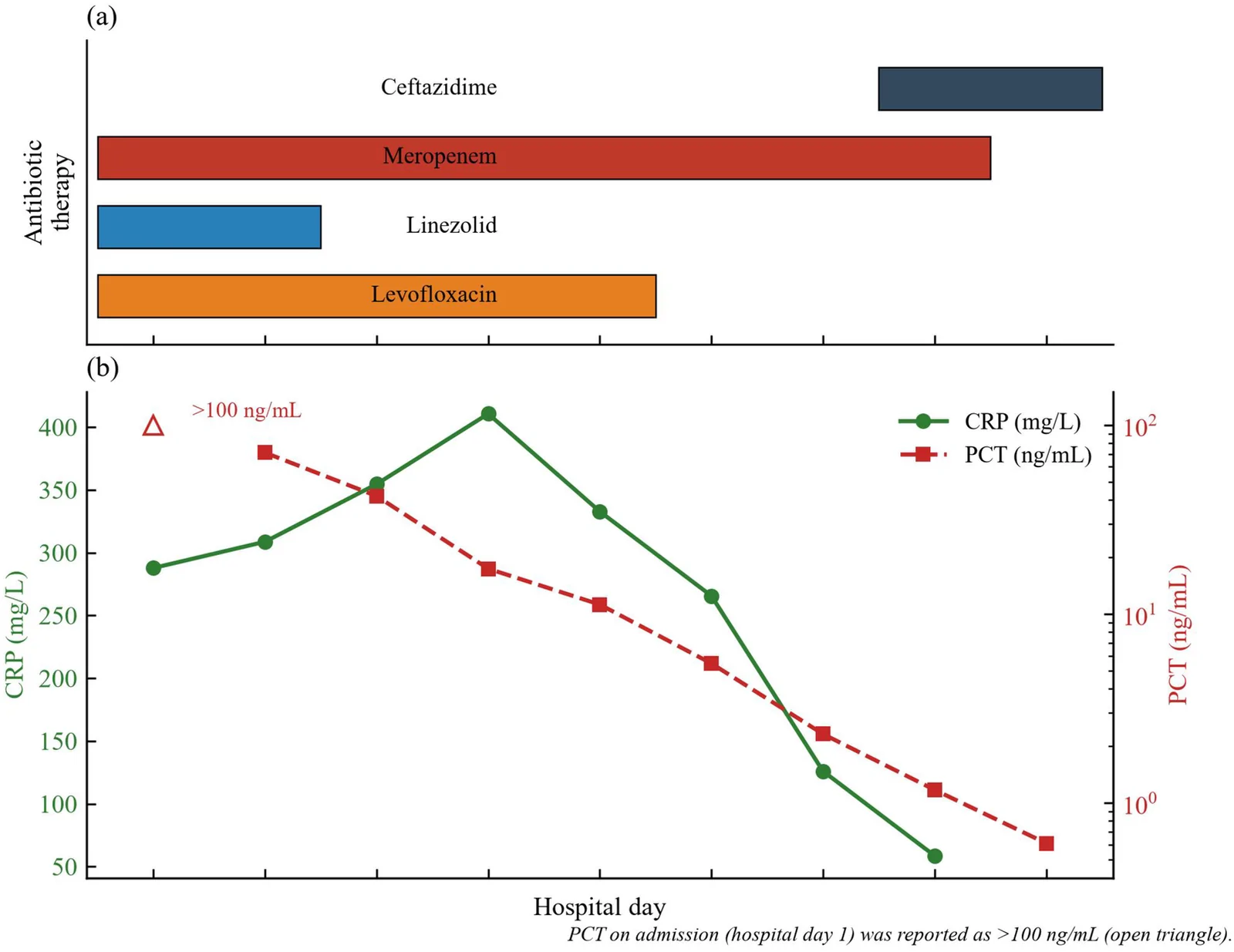
Serial C-reactive protein (CRP) and procalcitonin (PCT) levels and antibiotic therapy during the hospital course. **(a)** Antibiotic therapy timeline (hospital days 1–9); **(b)** CRP (left axis) and PCT (right axis) trends by hospital day. The open triangle indicates that PCT on admission (hospital day 1) was reported as >100 ng/mL.

### Pathogen diagnosis

Given the rapidly progressive critical condition and unknown pathogen, peripheral blood was collected on the day of admission for metagenomic next-generation sequencing (mNGS) and bacterial culture; no tissue culture was performed. mNGS is a routine clinical service of the molecular laboratory of our hospital's Department of Laboratory Medicine and can be ordered by attending physicians for critically ill patients. DNA and RNA metagenomic sequencing was performed on the MGI high-throughput sequencing platform, which covers 17,913 bacterial species (including 331 mycobacterial and 338 mycoplasma/chlamydia/spirochete/rickettsial species), 10,265 viral species, 2,227 fungal species and 383 parasitic species; all quality-control parameters, including Q30, passed. In the peripheral blood sample, mNGS identified 358 sequence reads uniquely mapping to Vibrio vulnificus (relative abundance 51.20% within the Vibrio genus), together with low read counts of Escherichia coli (39 reads), Bacteroides stercoris (3 reads) and JC polyomavirus (9 reads), which were considered low-abundance bystander or commensal organisms or a latent virus without clear pathogenic significance in this clinical setting. On the basis of this result, the antimicrobial regimen was adjusted to targeted anti-infective therapy. On the fifth hospital day, the blood culture obtained on admission was positive for V. vulnificus, identified by matrix-assisted laser desorption/ionization time-of-flight mass spectrometry (MALDI-TOF MS), confirming the final diagnosis of V. vulnificus-induced necrotizing fasciitis combined with septic shock. The mNGS result was therefore available approximately 3 days earlier than the culture-based confirmation. The clinical timeline is summarized in Table 1.

**Table 1:** Timeline of the clinical course.

Hospital day	Key clinical events
Day 1	Emergency department presentation with rapidly progressive swelling, erythema and subcutaneous ecchymosis of the right lower limb; ICU admission; initiation of empiric broad-spectrum antibiotics; emergency fasciotomy, radical debridement and vacuum sealing drainage (VSD); sampling of peripheral blood for culture and mNGS
Day 2	mNGS result positive for V. vulnificus (358 reads); antimicrobial regimen adjusted to targeted therapy
Day 3	Norepinephrine gradually weaned off
Day 4	Second surgery with complete resection of all necrotic tissue; continued VSD negative-pressure management
Day 5	Extubation and switch to nasal cannula oxygen; blood culture positive; V. vulnificus identified by MALDI-TOF MS
Day 8	Targeted intravenous ceftazidime (2 days) administered during the final two hospital days
Day 9	Discharge and transfer to local hospital for continued VSD dressing changes
6-month follow-up	Complete recovery of normal walking function; no limb dysfunction or recurrent infection

## Discussion

Clinical Characteristics of Atypical Occult V. vulnificus Infection

Most domestic and international V. vulnificus infection cases recorded in previous literature have a clear aquatic exposure history, including raw seafood consumption or wound exposure to coastal seawater (1, 3). The patient and his cohabiting family members clearly stated no history of seafood consumption or seawater contact. This case has two atypical core features: the patient recalled no seafood or seawater contact, and he had no classic underlying high-risk comorbidities, which distinguishes it from conventionally reported cases. It should be acknowledged that the absence of aquatic exposure relied on the patient's self-report and cannot be definitively confirmed; unrecognized contact with contaminated aquatic products, contaminated domestic water or minor skin trauma cannot be fully excluded. Nevertheless, this case demonstrates that V. vulnificus infection should be considered even when no such exposure is recalled.

Several retrospective studies and sporadic case reports have documented inland V. vulnificus infection without recalled exposure clues. Potential occult transmission pathways include indirect contact with contaminated aquatic products in markets, minor unrecognized skin micro-trauma exposed to low-salinity domestic water, and rare insect vector transmission (4). Clinicians tend to exclude V. vulnificus when patients lack a recalled aquatic exposure history, which easily causes delayed differential diagnosis. This case highlights that V. vulnificus infection cannot be ruled out solely on the basis of a negative recalled epidemiological history, even in inland medical centers.

### Limitations of traditional diagnostic modalities

The early cutaneous manifestations of V. vulnificus necrotizing fasciitis lack specificity, manifesting only as local redness, swelling and pain, which are highly similar to ordinary bacterial cellulitis (7). The LRINEC scoring system, widely used to screen necrotizing soft tissue infection, exhibits poor diagnostic sensitivity for V. vulnificus infection and often produces false-negative results, delaying early intervention (5). In the present case, the LRINEC score on admission was 4, below the widely used threshold of 6, yet the patient developed fulminant necrotizing fasciitis and septic shock, further illustrating the limited diagnostic sensitivity of this scoring system for V. vulnificus infection. Although bacterial culture remains the diagnostic gold standard, its long incubation cycle of 48–72 h cannot satisfy the diagnostic demands of shock patients whose condition deteriorates within hours. In addition, preoperative empirical antibiotics will further reduce the positive culture rate, bringing greater diagnostic obstacles (6). Extremely elevated PCT (>100 ng/mL) observed in this case reflects the explosive systemic inflammatory response induced by V. vulnificus, which can serve as an auxiliary warning marker for severe infection (8).

### Potential diagnostic value of mNGS for occult severe infection

In this case, mNGS identified V. vulnificus on the second admission day, approximately 3 days before the positive culture results obtained on hospital day 5, providing direct evidence for targeted anti-infective treatment. Unlike culture methods restricted by antibiotic interference and incubation time, mNGS can directly detect all pathogenic nucleic acid sequences in clinical samples without bias, realizing ultra-early pathogen identification for unexplained fulminant sepsis (6). For rare, atypical infections lacking epidemiological hints, mNGS fills the diagnostic gap of traditional microbial testing and creates a valuable window for early precise treatment.

### Core treatment principles and clinical enlightenment

Existing clinical consensus confirms that delayed surgical debridement is the primary risk factor for poor prognosis of V. vulnificus necrotizing fasciitis (9). In this case, emergency fasciotomy and decompression were performed immediately after ICU admission before definitive pathogen diagnosis, strictly following the core strategy of “early recognition, early surgical intervention”. Repeated debridement combined with VSD negative-pressure drainage thoroughly removed necrotic tissue, blocked the continuous release of bacterial toxins, and laid a foundation for subsequent skin graft reconstruction and limb salvage (3). Multidisciplinary collaboration covering emergency, ICU, infectious diseases and plastic surgery, including timely surgical intervention, targeted antibiotics and full organ support, is the key to reducing mortality of severe V. vulnificus infection.

### Clinical warning significance

This atypical case delivers two important clinical reminders:

For patients presenting with rapidly progressive limb swelling, subcutaneous hemorrhage, bullae and concurrent septic shock, clinicians must include V. vulnificus in differential diagnosis regardless of whether there is seafood or seawater exposure history;

mNGS should be prioritized for pathogen detection in critical patients with unknown source severe sepsis, especially when traditional culture and scoring systems cannot support a clear diagnosis, to shorten diagnostic time and optimize therapeutic schemes.

## Conclusion

For patients suffering from rapidly progressive necrotizing fasciitis complicated with refractory septic shock, clinicians should not exclude Vibrio vulnificus infection merely because of the absence of a recalled aquatic exposure. mNGS provides vital technical support for early and accurate identification of occult aggressive marine bacterial pathogens. Under a multidisciplinary team treatment framework, adherence to the principle of “early diagnosis, early radical debridement and timely targeted antibiotics” is the core measure to improve survival and limb salvage rates of V. vulnificus-infected patients.

## Data Availability

The raw data supporting the conclusions of this article will be made available by the authors, without undue reservation.
